# The degree of urbanisation reduces wild bee and butterfly diversity and alters the patterns of flower-visitation in urban dry grasslands

**DOI:** 10.1038/s41598-023-29275-8

**Published:** 2023-02-15

**Authors:** Johann Herrmann, Sascha Buchholz, Panagiotis Theodorou

**Affiliations:** 1 Department of Ecology, TU Berlin, Rothenburgstraße 12, 12165 Berlin, Germany; 2 Institute for Bee Protection, Julius Kühn Institute (JKI) - Federal Research Centre for Cultivated Plants, Messeweg 11/12, 38104 Braunschweig, Germany; 3 Institute of Landscape Ecology, University of Münster, Heisenbergstraße 2, 48149 Münster, Germany; 4 General Zoology, Institute for Biology, Martin-Luther University Halle-Wittenberg, Hoher Weg 8, 06120 Halle (Saale), Germany; 5 German Centre for Integrative Biodiversity Research (iDiv) Halle-Jena-Leipzig, Puschstraße 4, 04103 Leipzig, Germany

**Keywords:** Biodiversity, Community ecology, Conservation biology, Ecological networks, Urban ecology

## Abstract

Insect-provided pollination services are increasingly threatened due to alarming declines in insect pollinator populations. One of the main threats to insect pollinators and consequently pollination is urbanisation. Here, we investigate the effects of local habitat quality (patch size, flowering plant richness, bare soil cover, vegetation structure), degree of urbanisation (impervious surfaces) and 3D connectivity on bee, hoverfly and butterfly flower visitors and plant-flower visitor networks in flower-rich urban dry grasslands. Overall, the degree of urbanisation and the quality of the local habitat influenced the flowering plant and pollinator communities. Although flowering plant abundance increased with urbanisation, bee species richness and butterfly species richness decreased with increasing impervious surfaces. Flowering plant richness and ground nesting resource availability were positively related to bee richness and local vegetation structure boosted hoverfly and butterfly visitation rates. In terms of plant–pollinator interactions, insect pollinators visited a lower proportion of the available flowering plants in more urbanised areas and network modularity and specialisation increased with patch size. Our findings show that urban dry grasslands are valuable habitats for species-rich pollinator communities and further highlight the importance of minimizing the intensity of urbanisation and the potential of local management practices to support insect biodiversity in cities.

## Introduction

Due to alarming declines in insect populations^1–3^, insect-provided ecosystem services are increasingly threatened^4,5^. One of the most prominent and severely affected insect-provided ecosystem service is pollination^6–8^. Insect pollinators play a key role in almost all terrestrial ecosystems, as they are responsible for the reproduction of most wild flowering plants and global food crops^8–10^. The main causes of recent pollinator declines are habitat loss, fragmentation and degradation^6,7,11^. These processes are closely linked to intensified agriculture and urbanisation^6,11,12^.

Although intensified agricultural practices (e.g. use of pesticides and monocultures) are recognised as threats to all insects^6,11,13^, the effects of urbanisation on insect pollinators are more ambiguous^12^. Urban areas are characterised by a high degree of habitat loss and fragmentation and are often associated with numerous environmental stressors (e.g. urban heat island, air, light, water and soil pollution) with overall negative effects on insect diversity^14^. At the same time, cities have enormous habitat structural diversity, which can lead to a generally high biodiversity^15,16^. Furthermore, some urban green land uses can offer high availability of floral resources^17^, continuity of floral resources and novel nesting opportunities and thus support high insect pollinator diversity^18^. Overall, the influence of urbanisation on insect pollinators is complex and depends on the geographic region, spatial scale of investigation, taxonomic group studied and intensity of urban stressors^12,19,20^.

Among insects, wild bees are the most important group of pollinators due to their strong dependence on nectar and pollen for food^9,10,21^, but other non-bee insects such as hoverflies and butterflies are also frequent flower visitors and effective pollinators of many wild flowering plants and crops^22,23^. Furthermore, hoverflies contribute to long-distance pollen transfer with beneficial consequences for plant population health^22^. Although many wild bee species can find good refuge habitats in moderately urbanised environments^18,20,25^, many hoverfly^24–26^ and butterfly species do not find their necessary habitat requirements in cities^20,27,28^.

Compared to the number of studies investigating the effects of urbanisation on the diversity and species composition of pollinator and plant communities, little is known about the effects of urbanisation on plant–pollinator interactions and mutualistic network architecture^29^. Previous studies have shown that plant–pollinator network specialisation increases^30^, decreases^26^ or does not change^31^ with increasing urbanisation. However, urban flower visitors in most studies were found to be more specialised and visited proportionally fewer flowering plant species^26,30,31^. Urbanisation was also reported to have mixed effects on interaction evenness, positive^32^ or negative^33^. Such contrasting findings might be due to the differences in how each study quantified urbanisation (e.g. urban gradient or land-use classification), as well as due to differences in the method used to quantify flower visitor interactions. Some studies sampled flower visitation data on standardized experimental flowering plant communities^32,33^, while others used the natural vegetation of their study sites^26,30,31,34^. Furthermore, studies differed in their taxonomic resolution. Some studies identified pollinators at the species level^26,30,34^ while others at morphogroups/morphospecies^31–33^.

In our study, we quantified plant–pollinator networks in urban dry grasslands to examine how local habitat (patch size, flowering plant richness, bare soil cover, vegetation structure), degree of urbanisation (impervious surfaces), and landscape connectivity (3D connectivity) could affect flowering plants, their bee, butterfly, and hoverfly flower visitor communities and their mutualistic interactions. Furthermore, we built networks at the species and genus levels and tested how network metrics at the respective taxonomic resolution level relate to local habitat and landscape variables. We focused on the following research questions: what are the effects of local habitat, degree of urbanisation and landscape connectivity on (i) local flowering plant and pollinator communities and (ii) mutualistic network architecture?

## Methods

### Study system and study area

We conducted our study during May to August 2021 in and near the administrative region of the city of Berlin. Berlin is one of Germany’s largest metropolitan regions, spanning 891.1 km^2^ with a population of approximately 3.6 million people. For site selection, we used the research platform CityScapeLab Berlin^35^. CityScapeLab Berlin is a research platform designed for the investigation of the effects of urbanisation on biodiversity. It comprises 56 study sites throughout Berlin and the surrounding area of the Federal State of Brandenburg. CityScapeLab Berlin uses urban dry grasslands as a model ecosystem, as urban grasslands are known to be an essential component of urban green spaces globally^35^. Urban dry grasslands represent optimal habitats for wild bees^36,37^, butterflies^36^ and are good foraging grounds for hoverflies^37^. For our study, we selected 11 dry grasslands of CityScapeLab Berlin, while considering the representation of an urban gradient and an even geographical distribution throughout the city (Fig. S1 and Table S1). The dry grasslands were exclusively characterised by spontaneous vegetation and therefore only harboured wild plant species. Typical of urban dry grasslands, the vegetation was dominated by non-native species^35^ such as *Berteroa incana* and *Medicago x varia*.

### Sampling flower-visitors and flower visitor networks

We sampled flower visitor interactions monthly at each site from May to August 2021 using two 30-min transect walks, one in the morning (09:00–12:00) and one in the afternoon (14:00–17:00). We recorded an interaction when a flower visitor touched the reproductive parts of a flower. We sampled only during good weather conditions for insect pollinator activity (> 15 °C, wind speed less than 8 ms^−1^, no rain, sunny with clear skies). The sampling was performed by the same collector. We identified most butterflies at the species level in the field using the local identification literature^38^ (Table S2). We dried, pinned and identified all other insect flower visitors at the species level using identification keys^39–48^ (Tables S3 and S4) and DNA barcoding of the cytochrome oxidase I (COI) gene (Supplementary methods). Sequences obtained from COI barcoding were submitted to the NCBI GenBank database (Accession Numbers OP594212-OP594242). The insect specimens are currently deposited in the research collection of the first author (J. Herrmann). We identified all flowering plants directly in the field at the species level using taxonomic identification keys for the local flora^49^ (Table S5). We performed the data collection in accordance with the relevant guidelines and regulations.

### Landscape variables

We used the percentage of impervious surfaces and 3D connectivity as predictor variables (Table 1) for insect pollinator and flowering plant community structure and flower visitor network architecture. The percentage of impervious surfaces is an important predictor of pollinator community structure within cities^12^. We estimated the percentage of impervious surfaces at each site at three spatial scales; 100 m, 500 m and 1000 m^50^. To determine the spatial scale at which impervious surfaces had the most power to explain insect pollinator and flowering plant community structure and flower visitor network architecture, we correlated each observed variable with impervious surfaces at 100 m, 500 m and 1000 m scales and compared the correlation coefficients. We selected the scale with the highest correlation coefficient between impervious surfaces and the respective response variable for downstream statistical analyses^51^ (Table S6). We applied this method to each response variable to account for the dispersal abilities and habitat requirements of the different groups of organisms used in our study^51^.

**Table 1:** List of the environmental predictors used in this study.

	Predictor variables
	Impervious surfaces Percentage of impervious surfaces within a 100 m, 500 m or 1000 m radius around each study site^50^
	3D connectivity Modified Hanski’s habitat connectivity index^53,54^: connectivity metric that considers area size, distance to other dry grassland patches and building heights^52,55^
	Patch size Size of dry grassland patch^52^
	Flowering plant richness—local floral resource diversity Number of flowering plant species for each study site recorded using 8 1-m^2^ quadrats
	Vegetation height—local habitat structure for hoverflies and butterflies Average vegetation height in July recorded using 8 1-m^2^ quadrats
	Bare soil cover—nesting availability for wild bees Average amount of bare soil cover recorded using 8 1-m^2^ quadrats
	Honey bee abundance Total number of honey bee flower visits at a study site

Since insect pollinators use airspace, we used a 3D connectivity measure^25,37^, which incorporates building heights within the cityscape. This connectivity measure considers the distance to other dry grassland biotopes^52^, their size and building heights to represent 3D connectivity^35^ and was previously found to be an important predictor of urban pollinator communities^25,37^. The 3D-connectivity measure is based on the Hanski‘s habitat connectivity index^53,54^. We modified the distance-weighting factor that originally describes a species dispersal capacity to take in consideration the 3D cityscape context. For this, we summed the heights of buildings^55^ in corridors of 25 m radii around the connecting routes between patches. Thus, with a higher and increasing number of buildings between patches the distance increases, which reflects a reduction in connectivity and increased isolation. We contacted all spatial analyses in QGIS v.2.18.11 using the tools Edge distance vector of the Conefor Inputs plugin^56^ and Zonal statistics.

### Local habitat variables

While performing our transect walks, we also quantified several local habitat variables that could influence insect pollinators and flower visitor networks. During each sampling round, we used eight randomly placed 1-m^2^ quadrats and (1) quantified the percentage of bare soil cover as a surrogate of nesting resource availability for ground nesting bees, which make up a large part of the local wild bee fauna^57^ and (2) recorded all herbaceous plant species in flower (flowering plant richness—#), their coverage (flowering plant abundance—%), their number of flowering units (#) and the maximum vegetation height of flowering plants, grasses and non-flowering herbaceous plants (cm). Vegetation height illustrates the local habitat structure for hoverflies and butterflies. Many species of both pollinator groups depend on plant structures for food and habitat, especially at the larval stage^58,59^. We identified all flowering plants directly in the field at the species level using identification keys for the local flora^49^ (Table S7). We performed the data collection in accordance with the relevant guidelines and regulations. Furthermore, we quantified patch size (size of the dry grassland biotope) for each dry grassland studied^52^ using QGIS v.2.18.11.

### Flower visitor network architecture

For flower visitor network analysis, we used pooled data from all sampling rounds. We estimated network metrics using the R package bipartite v.2.16^60^. For each plant-flower visitor network, we calculated five commonly used network metrics that describe central aspects of network structure and are considered relevant for biodiversity conservation^61^: connectance, nestedness (NODF), modularity, network specialisation (H2’) and flower visitor specialisation (d′). Connectance describes the proportion of realised interactions within a network and is calculated as the sum of links divided by the number of cells in the matrix^60^. Nestedness (NODF) portrays whether specialised species (with fewer partners) tend to interact with subsets of more generalised species (more-connected species)^62^. Modularity describes a structural aspect of networks in which certain parts of a network are more interconnected than others. We used the QuanBiMo algorithm (R package bipartite v.2.16) to calculate modularity^63^. Network level specialisation describes the degree of niche divergence among species and is scaled between 0 (highly generalised) and 1 (highly specialised)^64^. Flower visitor specialisation (d′) reflects how specialised a species is considering the available floral resources and ranges from 0 (highly generalised) to 1 (high specialisation)^64^. We calculated overall flower visitor specialisation (d′) by averaging the d′ value for each flower visitor species per network. Since network metrics depend heavily on network size^60^, we used the Patefield’s algorithm^65^ and simulated 1000 random interaction networks (100 for modularity) for each site to then ∆-transform (standardise) all network metrics^66,67^. We calculated the ∆-transformation with $N-\overline{{N}_{r}}$ where $N$ is the observed value of a network metric and ${\overline{N}}_{r}$ is the mean value for the 1000 randomised networks and it reflects the extent to which a network metric differs from random expectations. Due to the potential influence of taxonomic resolution on mutualistic network metrics^68^, we calculated the ∆-transformed network metrics for the species and genus level (Table S8).

### Statistical analysis

To investigate the effects of impervious surfaces, 3D connectivity and local habitat variables (patch size, flowering plant richness, bare soil and vegetation height) on flower visitation rates as well as species richness and Shannon diversity of wild bees, hoverflies and butterflies we used generalised linear models (GLMs) or linear models (LMs) depending on the data type (i.e. for count data we used GLMs and for numerical data we used LMs). We applied three separate models for each wild pollinator group using their visitation rates (GLM), species richness (GLM) and Shannon diversity (LM) as response variables. Additionally, we applied a GLM to test the effects of impervious surfaces, 3D connectivity and local habitat variables on overall visitation rates. Impervious surfaces, 3D connectivity and local habitat variables were used as predictor variables (Table 1). Due to the potential effect of honey bees on wild bee communities^69,70^, we used the abundance of honey bees per site as an additional predictor. Furthermore, we used a GLM to explore the effects of cityscape (impervious surfaces and 3D connectivity) on honey bee abundance.

We used GLMs and LMs to explore the relationship between impervious surfaces, 3D connectivity and patch size and local flowering plant community structure. We applied four models in which the number of flower units (GLM), the cover of flowering plant species (GLM), flowering plant richness (GLM) and Shannon diversity of flowering vegetation (LM) were used as response variables.

We checked each model for multicollinearity using variance inflation factors with a cut-off value of 3^71^ and reduced the predictor set if necessary. For count data, we applied Poisson models. In case of overdispersion, we used negative binomial error models. For Shannon diversity (normally distributed), we applied Gaussian models. To identify the most parsimonious model for insect pollinator and flowering plant community structure and flower visitor network architecture, we used an automated model selection approach based on the Akaike Informationc Criterion (AIC), using the dredge function (R package MuMIn) with a maximum of three predictors to avoid overfitting^72^. We tested the residuals of all models for spatial autocorrelation using Moran’s *I* implemented in the R package ape^73^. In the case of spatial autocorrelation, we used generalised least squares models (GLS). Patch size was log-transformed to meet model assumptions. We checked all model (GLMs, LMs and GLSs) assumptions (residuals normally distributed, homogeneity of variance, linearity, no-outliers) visually and found to conform to expectations.

To determine the effects of cityscape and local habitat variables on flower visitor community composition, we applied non-metric multidimensional scaling (NMDS). To avoid statistical noise, we only used pollinator species with three or more flower visits^25,74^. We used the Wisconsin double standardisation and square root transformation to standardise the relative abundances of pollinator species and the Bray–Curtis dissimilarity matrix of the pollinator community for scaling. To search for a stable solution, we used a maximum number of 100 random starts. We fitted environmental variables to the ordination with 99,999 permutations to assess their relationship with community composition.

We used LMs to analyse the relationships between network structure and impervious surfaces, 3D connectivity and local habitat variables. We also used honey bee visitation rates as a predictor. The network metrics of each network resolution level served as response variables. We checked each model for multicollinearity, used the dredge function for model selection and checked model residuals for spatial autocorrelation and other model assumptions. We used Pearson’s correlation to compare network metrics at species and genus levels. We performed all analyses in R statistical software v.4.1.1 (R Core Team 2021).

## Results

In total, we detected 2016 interactions between 166 pollinator species and 67 plant species, of which 1095 (54%) were performed by wild bees (Anthophila), 270 (13%) by hoverflies (Syrphidea) and 203 (10%) by butterflies (Papilionoidea). The remaining 448 (22%) interactions were performed by honey bees (*Apis mellifera*). Wild bees were represented by 105 species (63%), hoverflies by 38 species (23%) and butterflies by 22 species (13%). The network-independent vegetation mapping of flowering plants using quadrats resulted in the detection of 54 flowering plant species.

### Flower visitors and flowering plant community structure along an urbanisation gradient

**Figure 1:**
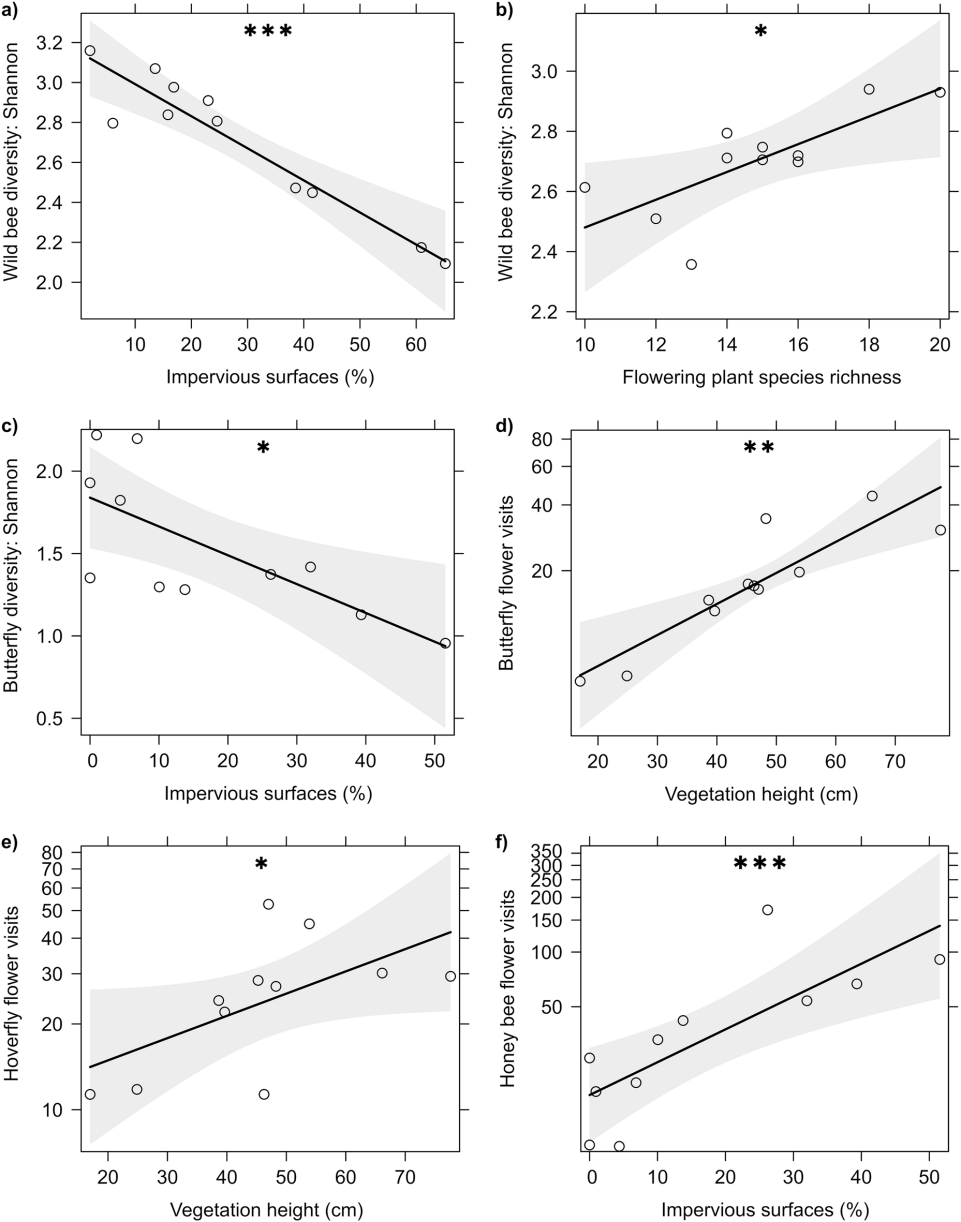
Relationships between (**a**) Shannon diversity of wild bees and impervious surfaces (1000 m); (**b**) Shannon diversity of wild bees and flowering plant richness; (**c**) Shannon diversity of butterflies and impervious surfaces (100 m); (**d**) butterfly flower visits and vegetation height; (**e**) hoverfly flower visits and vegetation height; (**f**) honey bee flower visits and impervious surfaces (100 m). Plotted lines show the predicted relationship and shaded areas indicate the 95% confidence intervals. *P < 0.05; **P < 0.01; ***P < 0.001.

The total number of pollinator visits was not affected by the degree of urbanisation (i.e. impervious surfaces), but increased in response to vegetation height (GLM; z = 2.039; P = 0.042; R^2^ = 0.246). The interactions and distribution patterns of individual pollinator groups differed along the urban gradient (Table 2). Impervious surfaces had a negative effect on wild bee species richness (GLM; scale: 500 m; z = − 2.375; P = 0.018; R^2^ = 0.298) and Shannon diversity of wild bees (LM; scale: 1000 m; t = − 6.029; P < 0.001; R^2^ = 0.841) (Fig. 1a). Flowering plant richness had a positive effect on the Shannon diversity of wild bees (LM; t = 2.731; P = 0.029; R^2^ = 0.841) (Fig. 1b). Due to multicollinearity with impervious surfaces, bare soil cover was not included in the model for wild bees. A separate model with bare soil cover revealed a positive, however not statistically significant influence of bare soil cover on wild bee species richness (GLM; z = 1.954; P = 0.051; R^2^ = 0.195). Honey bee visitation rates were positively related to the Shannon diversity of wild bees (LM; t = 3.172; P = 0.016; R^2^ = 0.841). Furthermore, impervious surfaces had a significant negative effect on the butterfly visitation rates (GLM; scale: 100 m; z = − 2.42; P = 0.016; R^2^ = 0.502), species richness (GLM; 100 m; z = − 2.373; P = 0.018; R^2^ = 0.558) and Shannon diversity (LM; 100 m; t = − 3.076; P = 0.013; R^2^ = 0.513) (Fig. 1c). The height of the vegetation had a positive effect on the butterfly visitation rates (GLM; z = 2.623; P = 0.009; R^2^ = 0.502) (Fig. 1d) and a positive effect on the hoverfly visitation rates (GLM; z = 2.209; P = 0.027; R^2^ = 0.261) (Fig. 1e). Impervious surfaces did not affect the number of flower visits or the diversity of hoverflies (GLM; P > 0.05). Honey bee flower visits increased with the degree of urbanisation (GLM; scale: 100 m; z = 3.874; P < 0.001; R^2^ = 0.516) (Fig. 1f).

**Table 2:** The most important predictors of pollinator and flowering plant community structure.

Response	Predictor				
	Impervious surfaces	Flowering plant richness	Vegetation height	Bare soil	Honey bee abundance
Total pollinator visitation rates			(+) P = 0.042		
Wild bees					
Species richness	(−) P = 0.018			(+) P = 0.051	
Shannon diversity	(−) P < 0.001	(+) P = 0.029			(+) P = 0.016
Hoverflies					
Visitation rate			(+) P = 0.027		
Butterflies					
Visitation rate	(−) P = 0.016		(+) P = 0.009		
Species richness	(−) P = 0.018				
Shannon diversity	(−) P = 0.013				
Honey bee flower visits	(+) P < 0.001				
Flowering plants					
Flower units	(+) P < 0.001				
Cover	(+) P < 0.001				
Shannon diversity	(−) P = 0.047

The degree of urbanisation had a positive effect on the number of flower units (impervious surfaces; GLM; scale: 1000 m; z = 3.579; P < 0.001; R^2^ = 0.578) and the coverage of flowering plants (GLM; scale: 500 m; z = 4.145; P < 0.001; R^2^ = 0.673). However, impervious surfaces had a negative effect on the Shannon diversity of flowering plants (LM; scale: 100 m; t = − 2.307; P = 0.047; R^2^ = 0.372).

**Figure 2:**
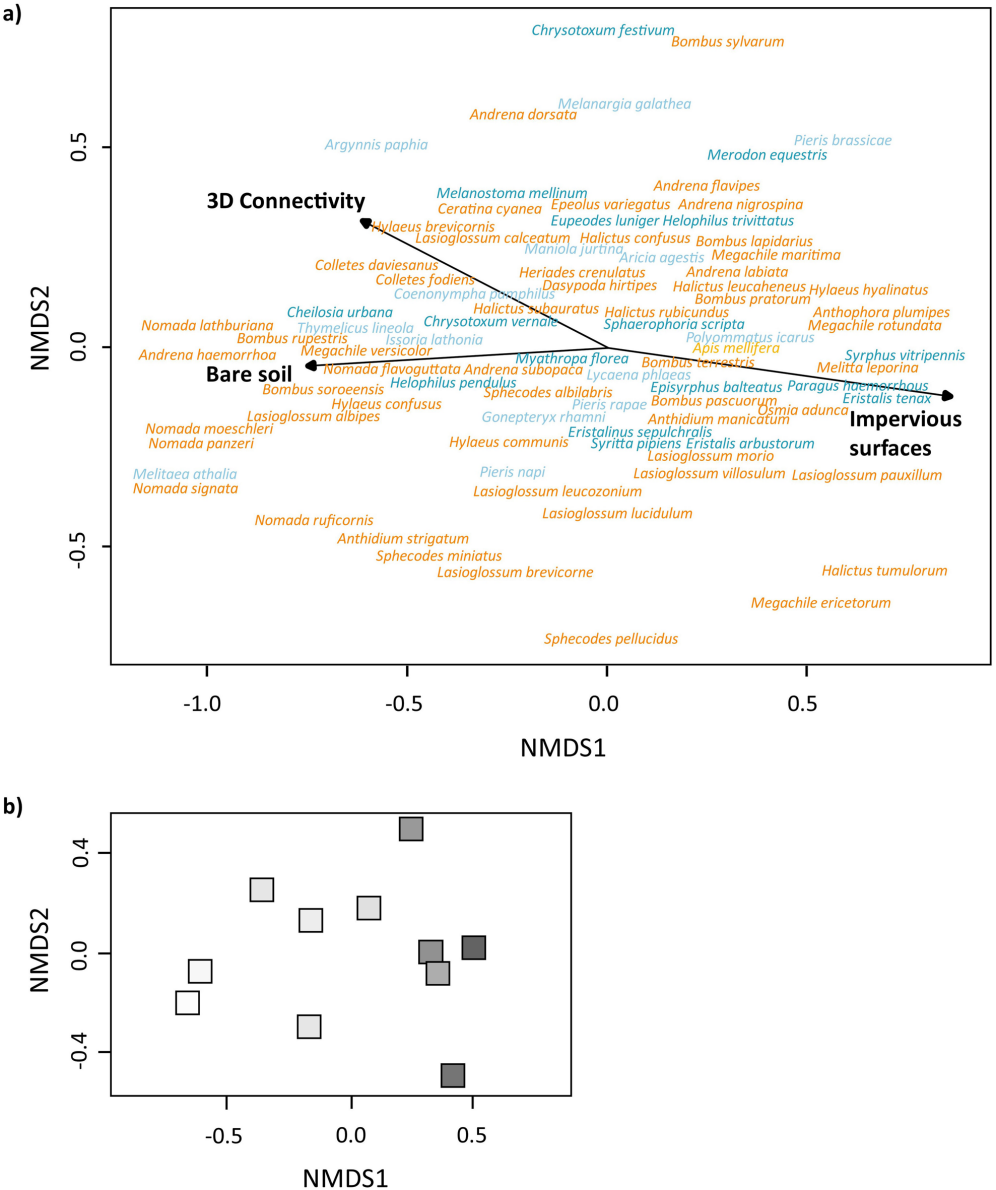
(**a**) Non-metric multidimensional scaling (NMDS) ordination of overall pollinator communities (stress value: 0.062). Impervious surfaces (500 m; P = 0.003), 3D Connectivity (P = 0.043) and the amount of bare soil (P = 0.034) are important factors that structure pollinator communities. Wild bee species are displayed in orange, hoverflies in dark blue, butterflies in light blue and honey bees in yellow. (**b**) The structuring of pollinator communities by impervious surfaces is also reflected in the positioning of the study sites in the NMDS-plot. The colour of the study sites indicates the degree of urbanisation (light grey: low proportion of impervious surfaces; dark grey: high proportion of impervious surfaces).

The NMDS analyses (stress value = 0.062) revealed that the composition of pollinator communities was significantly affected by impervious surfaces (500 m; P = 0.003; R^2^ = 0.817), 3D connectivity (P = 0.043; R^2^ = 0.506) and bare soil cover (P = 0.034; R^2^ = 0.616) (Fig. 2).

### Flower visitor network structure along an urbanisation gradient

**Figure 3:**
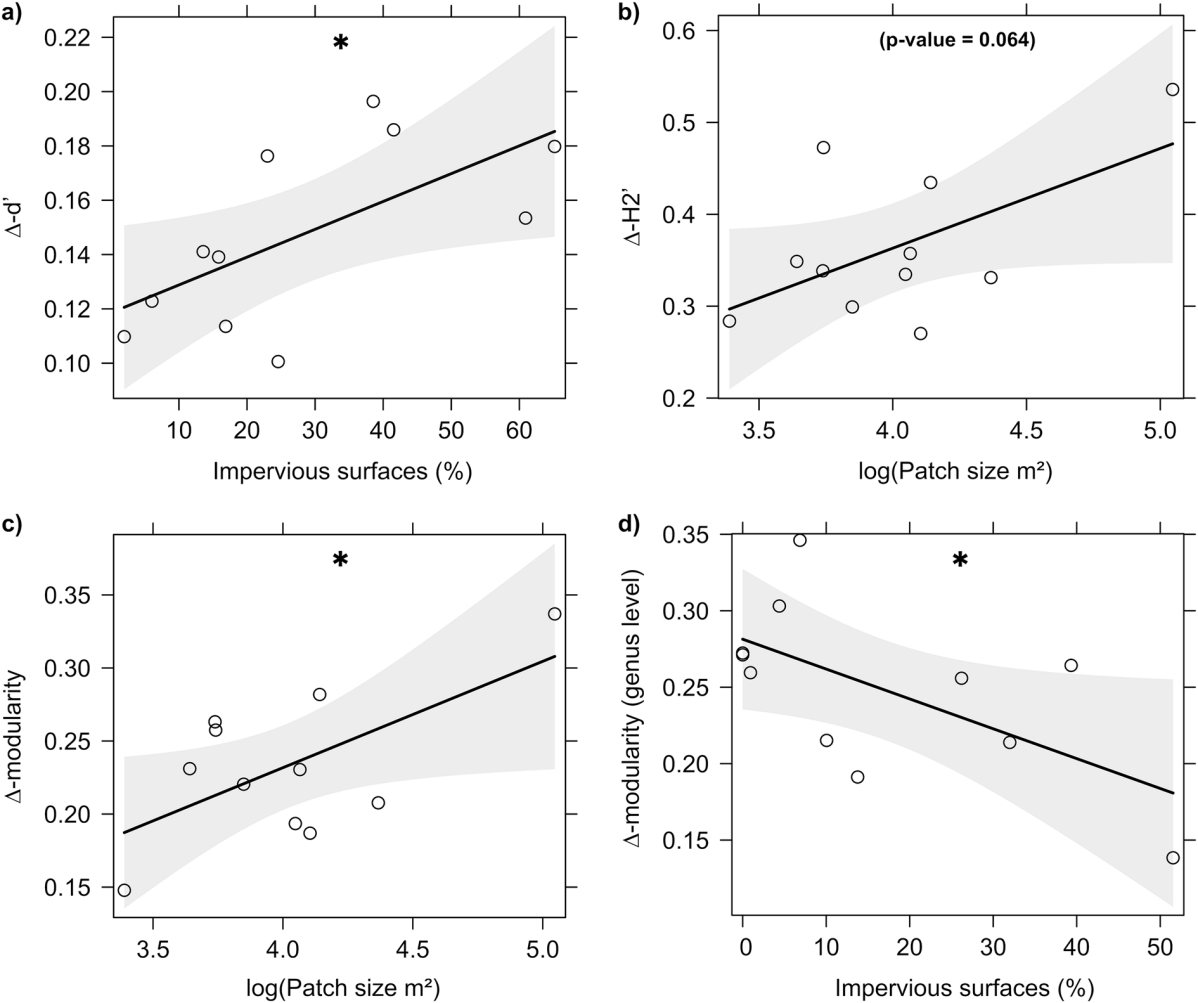
Relationships between (**a**) flower visitor specialisation (∆-d´) and impervious surfaces (1000 m); (**b**) network specialisation (∆-H2´) and log-transformed patch size; (**c**) network ∆-modularity and log-transformed patch size; (**d**) network ∆-modularity (genus level) and impervious surfaces (100 m). Plotted lines show the predicted relationship and shaded areas indicate the 95% confidence intervals. *P < 0.05.

**Figure 4:**
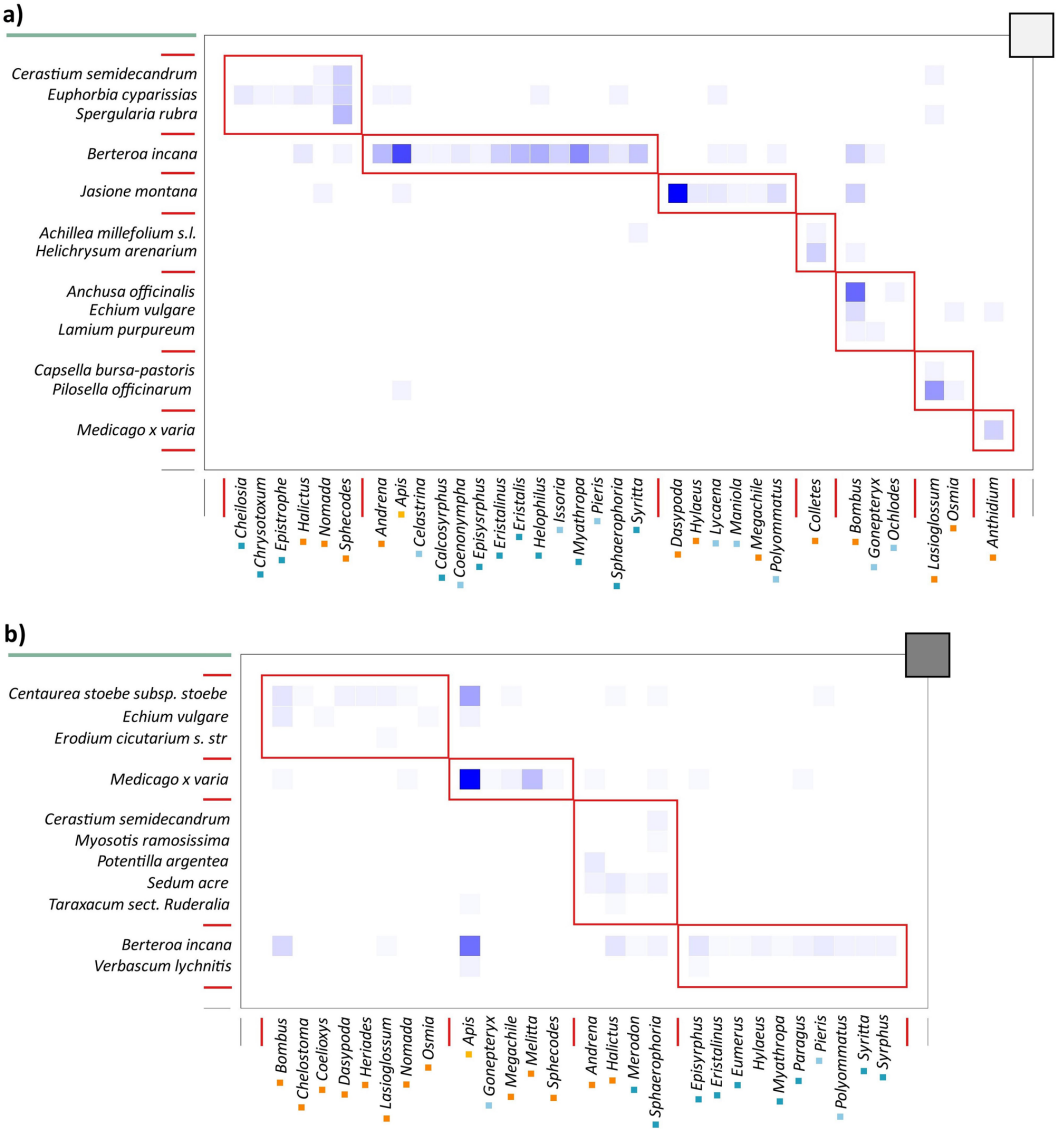
Comparison between two flower visitor networks (genus level) in terms of their modularity. (**a**) Modularity plot of a flower visitor network from a less urbanised site (site 1; ∆-modularity = 0.350). (**b**) Modularity plot of a flower visitor network from a highly urbanised site (site 6; ∆-modularity = 0.138). Flowering plants are resolved at the species level; pollinators at the genus level. Plants and pollinators are sorted by modular affinity, plant species as rows and pollinator genera as columns. The darker squares represent more frequent interactions. The red boxes separate the modules and the cells inside the boxes are the interactions within the modules. Wild bee genera are labelled orange, hoverfly genera dark blue, butterfly genera light blue and honey bees yellow.

Flower visitor networks were differentially affected by our predictor variables depending on the taxonomic resolution of the network (Table 3). When flower visitors were identified at the species level, impervious surfaces had a positive effect on ∆-d′ (GLS; scale: 1000 m; t = 2.524; P = 0.033) (Fig. 3a). Furthermore, patch size had a positive effect, although not statistically significant, on ∆-H2′ (LM; t = 2.112; P = 0.064; R^2^ = 0.331) (Fig. 3b) and on ∆-modularity (LM; t = 2.38; P = 0.041; R^2^ = 0.386) (Fig. 3c). When flower visitors were identified at the genus level, impervious surfaces had a negative effect on network ∆-modularity (LM; scale: 100 m; t = − 2.295; P = 0.047; R^2^ = 0.369) (Figs. 3d and 4). At both resolution levels, all the ∆-transformed network metrics differed from zero (t-test, P < 0.001). ∆-connectance and ∆-nestedness (NODF) were lower than zero, suggesting that all networks were less connected and nested than expected by chance. ∆-modularity was higher than zero, suggesting that all networks were more modular than expected by chance.

**Table 3:** The most important predictors of flower visitor network metrics.

Response	Predictor	
	Impervious surfaces	Patch size
Species level		
∆-d′	(+) P = 0.033	
∆-H2′		(+) P = 0.064
∆-modularity		(+) P = 0.041
Genus level		
∆-modularity	(−) P = 0.047

Except for ∆-NODF (r = 0.506, P = 0.112), most of network metrics at the flower visitor species or genus levels were highly correlated (∆-d′, r = 0.714, P = 0.014; ∆-H2′, r = 0.916, P < 0.001; ∆-connectance, r = 0.947, P < 0.001 and ∆-modularity, r = 0.878, P < 0.001). The values of ∆-d′, ∆-modularity, ∆-H2′ and ∆-NODF did not differ between networks at species or genus levels (t-test, P > 0.05). ∆-connectance, was higher in the networks at the species level (t-test, t = 2.175, P = 0.044).

## Discussion

In this study, we quantified plant–pollinator networks in urban dry grasslands and investigated how local habitat, degree of urbanisation and 3D connectivity might affect flowering plants and flower visitor communities and the structure of their mutualistic interactions. Overall, pollinator visitation rates were not affected by the degree of urbanisation, but total pollinator activity increased at sites with high vegetation height. Although we did not find an effect of impervious surfaces on hoverfly diversity, wild bees and butterflies were negatively affected by the degree of urbanisation. Furthermore, local flowering plant richness and bare soil cover had a positive effect on the diversity of wild bees and the height of vegetation had a positive effect on the visitation rates of butterflies and hoverflies. The community composition of pollinator species was influenced by the degree of urbanisation, bare soil cover and 3D connectivity. The degree of urbanisation had a positive effect on the abundance of flowering plants, but a negative effect on the Shannon diversity of flowering plants. In addition to the environmental effects on flower visitors, patch size and degree of urbanisation influenced the structure of flower visitation networks. We documented an increase in network specialisation and modularity with increasing patch size and along our urbanisation gradient insect pollinators visited a lower proportion of the available flowers.

Vegetation height positively influenced overall pollinator visitation rates. With increasing vegetation height, which is most likely associated with site management and lower mowing frequency, pollinator visitation rates increased. Our results are in line with previous studies which argue that flying insects benefit from lower mowing frequencies and higher vegetation structures^75^. Although overall insect pollinator visitation rates were not influenced by the degree of urbanisation, the effects of urbanisation on species diversity varied among taxonomic groups.

Bee diversity was negatively affected by the degree of urbanisation. Numerous previous studies have identified urban green land-uses as refuge habitats for wild bees affected by agricultural intensification^20,24,26^, while others, such as the present study, have detected negative effects of urbanisation on wild bee diversity^76,77^. Such contrasting findings could be due to methodological differences in the design of the studies^12^. Some studies compared urban vs. rural ecosystems while others have used a rural-to-urban gradient or an urbanisation gradient within city limits. Our study is consistent with previous findings performed along urbanisation gradients^76–78^. The increase in impervious surfaces results in a reduction of the available habitat for pollinators with negative consequences for individuals, populations and species^79,80^. While impervious surfaces had a negative effect on bees, local floral resource availability and increasing ground nesting resources had positive effects on bee richness. Bees depend on floral resources for food largely in the form of nectar and pollen and many studies have documented a strong relationship between bee and flowering plant diversity^81–83^. Bare soil cover for ground nesting bees was a good predictor of wild bee diversity, as previously reported for bee communities in rural and urban ecosystems^83,84^. This suggests that the negative effects of urban densification on wild bee diversity can be mitigated to some extent by increasing the availability of resources at the local patch level^82,83,85,86^. Increasing the diversity of floral food resources, for example, by establishing flower strips^87^, is a meaningful measure for the conservation of wild bees in cities.

Contrary to previous studies on hoverflies in urban contexts^24,26,88^, we did not detect a negative impact of urbanisation on hoverfly floral visitation rates and diversity. Hoverflies are flower generalists and, unlike wild bees, not central place foragers (not tied to a nest)^58^. Their general high mobility^22,58^ and unspecialised diet^22^ may allow many hoverflies to use spatially disconnected resources within the urban ecosystem. However, not all hoverfly species can thrive in cities. Many hoverfly species lack suitable habitats and resources for larval development (dung, ephermal water bodies, rotten wood, etc.) in urban areas^24,88^ and it is more likely that it is the presence rather than the accessibility of a resource that determines the occurrence of hoverfly species in cities^89^. This is also reflected in the low number of hoverfly species detected at our study sites (N = 38), which is only a fraction of the approximately 300 species recorded in the surroundings of Berlin^57^. Furthermore, 64% of all hoverfly interactions documented in our study were performed by common hoverfly species such as *Sphaerophoria scripta, Myathropa florea*, *Episyrphus balteatus*, *Helophilus trivittatus* and *Syritta pipiens*^58^. Nonetheless, hoverflies benefited from high vegetation. The increased activity of hoverflies associated with vegetation height^36^, may be related to the direct or indirect dependence of phytophagous and aphidophagous hoverfly species on plant structures for larval development^58^. Furthermore, high vegetation may increase the microhabitat structure and may influence the microclimate through shading. Within an urbanised environment, both aspects could be attractive to many hoverfly species since they are considered to prefer humid, structured and rather cooler habitats^58^.

As expected, based on previous findings, butterfly diversity was negatively affected by the degree of urbanisation^20,28,90,91^. Compared to wild bees that responded to the degree of urbanisation at large spatial scales (500 m and 1000 m), butterfly activity and diversity responded to the degree of urbanisation at a very small scale (100 m). Similarly, to our findings, Kuussaari et al.^28^ and Merckx and Van Dyck^90^ have also shown that urbanisation affects butterfly communities at very small scales (50 m) (Table S6). This may indicate a very strong local filtering effect in the structure of butterfly communities^92,93^. Similarly, to hoverflies, butterflies benefited from high vegetation^36,94^. Butterfly species require plant structures for larval development and therefore rely on habitats with a sufficient amount of plant biomass and vegetation structure^36,59^. Given the positive effects of vegetation height on hoverflies and butterflies as well as overall pollinator activity, reducing mowing frequency could increase the abundance of these pollinators and possibly overall pollinator activity in urban green land uses^75^.

In line with previous studies, the composition of the overall pollinator community was influenced at the landscape level by the degree of urbanisation and habitat connectivity^12,20,25,95^. Bare soil cover also had an influence on the overall flower visitor community composition, which can be explained by the high proportion of ground nesting bee species detected in our study (N = 59). Furthermore, the occurrence of parasitic species such as *Nomada lathburiana* (Apidae), *Nomada moeschleri* (Apidae), *Nomada panzeri* (Apidae) or *Nomada signata* (Apidae) was strongly associated with the availability of bare soil, which can be explained by their dependence on sufficiently large host populations of ground nesting *Andrena* species^25^.

Whereas urbanisation also predicted taxon-specific responses, 3D connectivity could only be identified as a predictor of overall community composition. This suggests that only individual species, rather than the studied taxa in general, are negatively affected by building heights or other barriers within the urban ecosystem (e.g. bees: *Hylaeus brevicornis* (Colletidae)*, Colletes daviesanus* (Colletidae), *Colletes fodiens* (Colletidae)*;* hoverflies: *Cheilosia urbana* (Syrphidae)*, Melanostoma mellinum* (Syrphidae)*;* butterflies: *Argynnis paphia* (Nymphalidae), *Thymelicus lineola* (Hesperiidae)). However, other species such as *Halictus tumulorum* (Halictidae)*, Lasioglossum pauxillum* (Halictidae)*, Lasioglossum morio* (Halictidae), *Melitta leporina* (Melittidae) for bees, *Eristalis arbustorum* (Syrphidae)*, Eristalis tenax* (Syrphidae)*, Paragus haemorrhous* (Syrphidae) for hoverflies and *Pieris rapae* (Pieridae), *Polyommatus icarus* (Lycaenidae) for butterflies seem to cope well in highly isolated and simultaneously highly urbanised areas. We assume that these species might be less dependent on 3D connectivity, if favourable factors such as floral resources, structurally rich vegetation for all life-cycle stages and bare soil are available at the local patch level^37^. This appears to be particularly the case for the rather small, ground nesting and social bee species *Halictus tumulorum* (Halictidae)*, Lasioglossum pauxillum* (Halictidae)*, Lasioglossum morio* (Halictidae)^25^. Social bee species can use resources and store food more efficiently and thus cope better in unfavourable conditions compared to solitary bee species^25,96^. In addition their rather small body size seems to facilitate their persistence in fragmented urbanised habitat patches^25^.

Consistent with previous findings, we observed a higher availability of floral resources with increasing urbanisation^17^. Because our study plots were entirely characterised by spontaneous vegetation, the increase in floral resources is not attributable to man-made plantings, as this has been the case in other studies that examined urban parks or gardens^17^. In highly urbanised areas, dry grasslands in our study were particularly characterised by non-native plant species such as *Berteroa incana* and *Medicago x varia*, which together accounted for 46% of the total number of floral units observed. The higher abundance of flowers observed in our highly urbanised sites can therefore be attributed to the presence of these non-native species, which have been shown to occur in large populations in cities and are often better adapted to urban environments^97,98^. Consequently, the presence of those flowering plants has implications for the occurrence of certain pollinator species. The oligolectic bee species *Melitta leporina* (Melittidae), which specialises on *Medicago* spp. was strongly associated with increasing urbanisation, which can be explained by the high abundance of *Medicago x varia* in our highly urbanised sites.

In addition to analysing the responses of wild pollinator and flowering plant communities to urbanisation, our study also investigated the potential effects of honey bee abundance on flower visitors^70,99^. Honey bees dominated flower visits at our study sites and were associated with increased urbanisation. Since honey bees are managed, their distribution probably mirrors the distribution of urban beekeepers in the city of Berlin. Surprisingly, we found a positive correlation between honey bee abundance and wild bee diversity. This result probably reflects the general attractiveness of flower-rich urban dry grasslands to both honey bees and wild bees. Future studies should quantify the abundance of honey bee hives within the urban ecosystem and investigate the potential influence of honey bees on urban pollinator biodiversity, health and pollination service provision.

All our Δ-transformed network metrics were consistently different from zero. Relative nestedness and connectance were lower than zero and relative modularity was higher than zero, suggesting the existence of isolated groups of interacting flowering plants and insect species/genera. In line with previous studies, pollinator species in highly urbanised areas were more specialised and visited proportionally less plant species from those locally available^26,30,31^. Although we did not find a relationship between patch size and individual flower visitor taxa, patch size had a positive influence on network specialisation and modularity. Modularity describes a central aspect of network structure and provides information on the stability and complexity of plant–pollinator communities and interaction networks^100–102^. High modularity reflects high community robustness, as disturbances and stressors are expected to spread more slowly through a modular network than a non-modular network^100,103^. The division into closely connected modules that are more independent from each other is most likely the result of increased functional and taxonomic diversity with increasing patch size^102,104,105^. Network specialisation is a measure of selectiveness and could indicate the level of functional redundancy and niche complementarity in a community^106^. Due to specialists being more dependent on larger areas, it is expected that network specialisation increases with increasing habitat area^107^. Indeed, in our study, we observed an increased network level specialisation with increasing patch size.

Furthermore, we observed changes in the statistical power to detect if certain environmental variables could affect network architecture by modifying the taxonomic resolution of our networks. While at the species resolution level we documented an effect of urbanisation and patch size on flower visitor and network specialisation and modularity, at the genus level we documented an effect of urbanisation on modularity. The reduction in modularity we observed with impervious surfaces is most likely due to the reduction of wild pollinator genera due to urban densification. Relative network metrics were sufficiently conserved up to the genus level (exception: ∆-NODF), suggesting that networks at the genus level could provide higher sensitivity to detect shifts in network modularity along environmental gradients.

As network structure and insect pollinator communities can vary significantly between years^108–110^, one potential limitation of our work is that our data come from a 1-year sampling. However, our study was not designed to exhaustively survey each site in multiple years; we rather aimed to sample multiple sites in 1 year using a standardised methodology to evaluate the effects of local and landscape features on flower visitor communities and on plant pollinator networks.

## Conclusions

Our findings reveal a negative effect of urban densification on the diversity of wild bees and butterflies in urban dry grasslands. Yet, we also observed that local resource availability and vegetation structure could have strong effects on overall pollinator activity, bee diversity and on butterfly and hoverfly visitation rates. Additionally, flower visitor networks were more modular and specialised in larger habitat patches, suggesting that plant–pollinator interactions in larger areas are more heterogeneous, probably reflecting higher functional diversity. From an applied perspective, the strong influence of local habitat quality on urban insect pollinators, points to the importance of (i) enhancing flowering plant richness by establishing species rich flower strips of sufficient size; (ii) reducing mowing regimes and tolerate high herbaceous vegetation on urban green land-uses and (iii) creating open sandy soil areas (e.g. sandarium) to provide nesting grounds for ground nesting bees as local management practices to support insect biodiversity conservation in cities.

## Supplementary Information

Supplementary Information.

## Data Availability

Data are available in Figshare: 10.6084/m9.figshare.21637208.v1.
