# Supplementary material for: The degree of urbanisation reduces wild bee and butterfly diversity and alters the patterns of flower-visitation in urban dry grasslands
Source: Sci Rep. 2023 Feb 15;13:2702. doi: 10.1038/s41598-023-29275-8 (PMC9932066; doi:10.1038/s41598-023-29275-8)
Supplement: Supplementary file 1 — Supplementary Information. [file 41598_2023_29275_MOESM1_ESM.pdf]

Supplementary material

The degree of urbanisation reduces wild bee and butterfly diversity and alters the patterns of flower-visitation in urban dry grasslands

Johann Herrmann[1,2*], Sascha Buchholz[1,3], Panagiotis Theodorou[4,5*]

[1] Department of Ecology, TU Berlin, Rothenburgstraße 12, 12165 Berlin, Germany

[2] Julius Kühn-Institute, Institute for Bee Protection, Messeweg 11/12, Braunschweig, Germany

[3] Institute of Landscape Ecology, University of Münster, Heisenbergstraße 2, 48149 Münster, Germany

[4] General Zoology, Institute for Biology, Martin-Luther University Halle-Wittenberg, Hoher Weg 8, 06120 Halle (Saale), Germany

[5] German Centre for Integrative Biodiversity Research (iDiv) Halle-Jena-Leipzig, Puschstraße 4, 04103 Leipzig, Germany

*Corresponding authors:

Johann Herrmann, email: johann.herrmann@posteo.de

Panagiotis Theodorou, email: panatheod@gmail.com

Supplementary figures

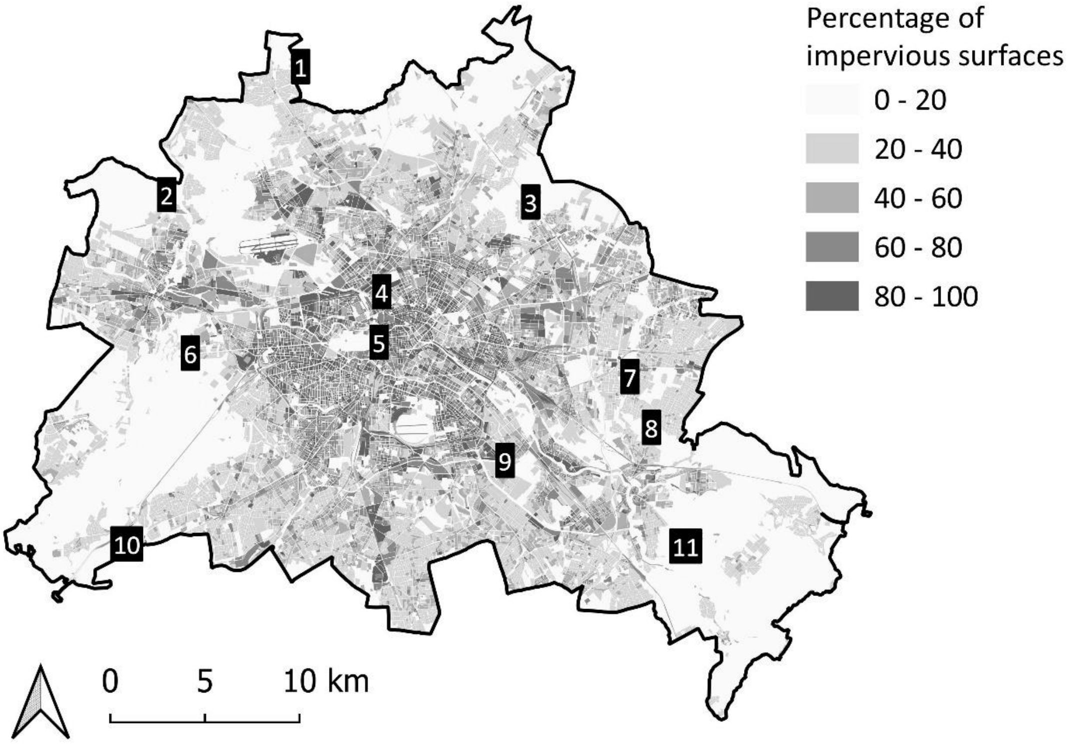

Figure S1. Study area and the 11 study sites in and near the administrative region of the city of Berlin (Germany). The percentages of impervious surfaces were used as a measure of urbanisation within a radius of 100 m, 500 m and 1000 m around the study plot (see main text, Methods and Table S1). Shades of grey correspond to percentages of impervious surfaces. This map was created with QGIS software version 3.16.10.[1]

Supplementary Tables

Table S1. Coordinates of the 11 study sites with information on impervious surfaces (%) within a radius of 100 m, 500 m and 1000 m, patch size (m^2) and bare soil cover (%).

Site	Long	Lat	Impervious surfaces 100 m radius (%)	Impervious surfaces 500 m radius (%)	Impervious surfaces 1000 m radius (%)	Patch size (m^2)	Bare soil cover (%)
1	13.30965	52.64434	6.844	10.744	13.560	111203.95	24
2	13.2075	52.58202	4.387	3.042	6.037	13850.92	23.38
3	13.4916	52.58245	10.048	11.893	15.832	23288.49	37.97
4	13.37698	52.53825	39.335	61.559	65.181	5476.94	1.13
5	13.37576	52.51426	13.764	54.402	60.913	12718.93	5.88
6	13.22902	52.50694	51.538	32.640	24.576	2452.21	1.84
7	13.57169	52.50037	31.986	40.502	38.543	4377.89	1.22
8	13.58918	52.47647	0.930	10.963	22.995	5509.15	24.59
9	13.47589	52.45935	26.210	43.206	41.544	11625.59	9.91
10	13.18328	52.41555	0	7.534	16.867	7060.25	26.16
11	13.61755	52.42034	0	1.500	1.968	11159.68	41.59

Table S2. List of butterfly species (Papilionidae) and their recorded number of flower visits.

Species	Flower visits
Aglais io (LINNAEUS, 1758)	1
Argynnis adippe (DENIS & SCHIFFERMÜLLER, 1775)	1
Argynnis paphia (LINNAEUS, 1758)	5
Aricia agestis (DENIS & SCHIFFERMÜLLER, 1775)	5
Celastrina argiolus (LINNAEUS, 1758)	1
Coenonympha pamphilus (LINNAEUS, 1758)	15
Gonepteryx rhamni (LINNAEUS, 1758)	11
Issoria lathonia (LINNAEUS, 1758)	17
Lycaena alciphron (ROTTEMBURG, 1775)	2
Lycaena phlaeas (LINNAEUS, 1758)	17
Lycaena tityrus (PODA, 1761)	1
Maniola jurtina (LINNAEUS, 1758)	18
Melanargia galathea (LINNAEUS, 1758)	8
Melitaea athalia (ROTTEMBURG, 1775)	5
Ochlodes sylvanus (ESPER, 1778)	2
Papilio machaon LINNAEUS, 1758	2
Pieris brassicae (LINNAEUS, 1758)	3
Pieris napi (LINNAEUS, 1758)	14
Pieris rapae (LINNAEUS, 1758)	44
Polyommatus icarus (ROTTEMBURG, 1775)	27
Thymelicus lineola (OCHSENHEIMER, 1808)	3
Vannessa cardui (LINNAEUS, 1758)	1
**Total**	203

Table S3. List of bee species (Anthophila) and their recorded number of flower visits.

Species	Flower visits
Andrena barbilabris (KIRBY, 1802)	2
Andrena bicolor (FABRICIUS, 1775)	1
Andrena cineraria (LINNAEUS, 1758)	2
Andrena denticulata (KIRBY, 1802)	1
Andrena dorsata (KIRBY, 1802)	5
Andrena flavipes PANZER, 1799	12
Andrena haemorrhoa (FABRICIUS, 1781)	8
Andrena helvola (LINNAEUS, 1758)	2
Andrena labiata (FABRICIUS, 1781)	3
Andrena minutula (KIRBY, 1802)	1
Andrena minutuloides (PERKINS, 1914)	1
Andrena mitis (SCHMIEDEKNECHT, 1883)	1
Andrena nigroaenea (KIRBY, 1802)	1
Andrena nigrospina (THOMPSON, 1872)	22
Andrena subopaca (NYLANDER, 1848)	26
Andrena ventralis (IMHOFF, 1832)	1
Andrena wilkella (KIRBY, 1802)	2
Anthidium manicatum (LINNAEUS, 1758)	7
Anthidium oblongatum (ILLIGER, 1806)	2
Anthidium strigatum (PANZER, 1805)	5
Anthophora bimaculata (PANZER, 1798)	1
Anthophora furcata (PANZER, 1798)	2
Anthophora plumipes (PALLAS, 1772)	29
Bombus bohemicus (SEIDL, 1837)	1
Bombus hortorum (LINNAEUS, 1761)	1
Bombus hypnorum (LINNAEUS, 1758)	1
Bombus lapidarius (LINNAEUS, 1758)	99
Bombus lucorum (LINNAEUS, 1761)	1
Bombus pascuorum (SCOPOLI, 1763)	126
Bombus pratorum (LINNAEUS, 1761)	4
Bombus ruderarius (MÜLLER, 1776)	1
Bombus rupestris (FABRICIUS, 1793)	10
Bombus soroeensis (FABRICIUS, 1776)	3
Bombus sylvarum (LINNAEUS, 1761)	5
Bombus terrestris (LINNAEUS, 1758)	134
Ceratina cyanea (KIRBY, 1802)	12
Chelostomma rapunculi (LEPELETIER, 1841)	1
Coelioxys conica (LINNAEUS, 1758)	1
Coelioxys conoidea (ILLIGER, 1806)	1
Coelioxys mandibularis (NYLANDER 1848)	1
Colletes daviesanus (SMITH, 1846)	27
Colletes fodiens (GEOFFROY, 1785)	36
Colletes marginatus (SMITH, 1846)	1
Dasypoda hirtipes (FABRICIUS, 1793)	53
Epeolus variegatus (LINNAEUS, 1758)	6
Halictus confusus (SMITH, 1853)	14

**Species**	**Flower visits**
Halictus leucaheneus (EBMER, 1972)	20
Halictus rubicundus (CHRIST, 1791)	15
Halictus sexcintus (FABRICIUS, 1775)	1
Halictus subauratus (ROSSI, 1792)	47
Halictus submediterranus (PAULY, 2015)	1
Halictus tumulorum (LINNAEUS, 1758)	6
Heriades crenulatus (NYLANDER 1856)	35
Heriades truncorum (LINNAEUS, 1758)	1
Hylaeus annularis (KIRBY, 1802)	2
Hylaeus brevicornis (NYLANDER 1852)	5
Hylaeus communis (NYLANDER 1852)	25
Hylaeus confusus (NYLANDER 1852)	5
Hylaeus hyalinatus (SMITH, 1842)	3
Lasioglossum aeratum (KIRBY, 1802)	1
Lasioglossum albipes (FABRICIUS, 1781)	3
Lasioglossum brevicorne (SCHENCK, 1868)	6
Lasioglossum calceatum (SCOPOLI, 1763)	14
Lasioglossum laticeps (SCHENCK, 1868)	2
Lasioglossum leucozonium (SCHRANK, 1781)	5
Lasioglossum lucidulum (SCHENCK, 1861)	16
Lasioglossum morio (FABRICIUS, 1793)	17
Lasioglossum pallens (BRULLÉ, 1832)	1
Lasioglossum pauxillum (SCHENCK, 1853)	31
Lasioglossum punctatissimum (SCHENCK, 1853)	1
Lasioglossum setulosum (STRAND, 1909)	1
Lasioglossum villosulum (KIRBY, 1802)	3
Megachile circumcincta (KIRBY, 1802)	2
Megachile ericetorum (LEPELETIER, 1841)	5
Megachile ligniseca (KIRBY, 1802)	2
Megachile maritima (KIRBY, 1802)	22
Megachile pilidens (ALFKEN, 1924)	1
Megachile rotundata (FABRICIUS, 1787)	3
Megachile versicolor (SMITH, 1844)	4
Melitta leporina (PANZER, 1799)	18
Nomada bifasciata (OLIVIER, 1811)	3
Nomada flavoguttata (KIRBY, 1802)	7
Nomada flavopicta (KIRBY, 1802)	1
Nomada fucata (PANZER, 1798)	1
Nomada goodeniana (KIRBY, 1802)	2
Nomada lathburiana (KIRBY, 1802)	3
Nomada moeschleri ALFKEN, 1913	7
Nomada panzeri (LEPELETIER, 1841)	6
Nomada ruficornis (LINNAEUS, 1758)	3
Nomada signata (JURINE, 1807)	3
Nomada striata (FABRICIUS, 1793)	1
Osmia adunca (PANZER, 1798)	22
Osmia anthocopoides (SCHENCK, 1853)	2
Osmia aurulenta (PANZER, 1799)	2

Species	Flower visits
Osmia bicornis (LINNAEUS, 1758)	1
Osmia leaiana (KIRBY, 1802)	2
Osmia spinulosa (KIRBY, 1802)	1
Panurgus calcaratus (SCOPOLI, 1763)	1
Sphecodes albilabris (FABRICIUS, 1793)	5
Sphecodes ephippius (LINNAEUS, 1767)	1
Sphecodes miniatus (VON HAGENS, 1882)	14
Sphecodes pellucidus (SMITH, 1845)	3
Stelis breviuscula (NYLANDER, 1848)	1
Tetralionella dentata (GERMAR, 1839)	2
Xylocopa violacea (LINNAEUS, 1758)	1
Apis mellifera (LINNAEUS, 1758)	448
**Total**	1543

Table S4. List of hoverfly species (Syrphidae) and their recorded number of flower visits.

Species	Flower visits
Chalcosyrphus nemorum (FABRICIUS, 1805)	1
Cheilosia aerea DUFOUR, 1848	2
Cheilosia urbana (MEIGEN, 1822)	3
Chrysotoxum festivum (LINNAEUS, 1758)	4
Chrysotoxum vernale LOEW, 1841	5
Dasysyrphus albostriatus (FALLÉN, 1817)	1
Didea intermedia LOEW, 1854	1
Epistrophe eligans (HARRIS, 1780)	1
Epistrophe melanostoma (ZETTERSTEDT, 1843)	2
Epistrophe nitidicollis (MEIGEN, 1822)	2
Epistrophella euchroma (KOWARZ, 1885)	2
Episyrphus balteatus (DE GEER, 1776)	27
Eristalinus sepulchralis (LINNAEUS, 1758)	7
Eristalis arbustorum (LINNAEUS, 1758)	6
Eristalis horticola (DE GEER, 1776)	1
Eristalis intricaria (LINNAEUS, 1758)	1
Eristalis tenax (LINNAEUS, 1758)	4
Eumerus strigatus (FALLÉN, 1817)	1
Eupeodes corollae (FABRICIUS, 1794)	2
Eupeodes luniger (MEIGEN, 1822)	4
Helophilus pendulus (LINNAEUS, 1758)	5
Helophilus trivittatus (FABRICIUS, 1805)	27
Melanostoma mellinum (LINNAEUS, 1758)	10
Merodon equestris (FABRICIUS, 1794)	7
Merodon moenium (WIEDEMANN, 1822)	2
Myathropa florea (LINNAEUS, 1758)	52
Neocnemodon pubescens (DELUCCHI & PSCHORN-WALCHER, 1955)	1
Paragus haemorrhous (MEIGEN, 1822)	4
Paragus spec.	2
Pipiza festiva (MEIGEN, 1822)	1
Pipiza noctiluca (LINNAEUS, 1758)	1
Scaeva pyrastri (LINNAEUS, 1758)	1
Sphaerophoria scripta (LINNAEUS, 1758)	55
Sphaerophoria taeniata (MEIGEN, 1822)	1
Syritta pipiens (LINNAEUS, 1758)	12
Syrphus ribesii (LINNAEUS, 1758)	1
Syrphus vitripennis (MEIGEN, 1822)	10
Xanthogramma dives (RONDANI, 1857)	1
**Total**	270

Table S5. List of plant species and the number of flower visits they received.

Species	Flower visits
Achillea millefolium s.l.	7
Ajuga genevensis	1
Anchusa officinalis	59
Arabidopsis arenosa	1
Arabidopsis thaliana	2
Arenaria serpyllifolia	3
Artemisia vulgaris	3
Berteroa incana	479
Capsella bursa-pastoris	1
Centaurea jacea	3
Centaurea stoebe subsp. stoebe	277
Cerastium arvense	16
Cerastium semidecandrum	29
Chondrilla juncea	12
Convolvulus arvensis	16
Crepis capillaris	2
Dianthus carthusianorum	9
Dianthus deltoides	7
Diplotaxis tenuifolia	7
Echium vulgare	150
Erigeron canadensis	4
Erodium cicutarium s. str	37
Euphorbia cyparissias	74
Galium verum	1
Geranium molle	2
Helichrysum arenarium	32
Hypericum perforatum	3
Hypochaeris radicata	6
Jasione montana	150
Knautia arvensis	4
Lamium purpureum	47
Malva moschata	1
Medicago lupulina	1
Medicago x varia	126
Myosotis ramosissima	6
Oenothera biennis s.l.	3
Ononis repens	16
Petrorhagia prolifera	5
Picris hieracioides	1
Pilosella officinarum	37
Plantago lanceolata	25
Potentilla argentea	59
Potentilla reptans	1
Potentilla verna	80
Salvia pratensis	1
Scorzoneroides autumnalis	1

**Species**	**Flower visits**
Sedum acre	9
Sedum rupestre	12
Sedum sexangulare	9
Senecio jacobea	1
Senecio vernalis	9
Solidago canadensis	5
Spergularia rubra	10
Tanacetum vulgare	53
Taraxacum sect. Ruderalia	6
Thymus pulegioides	24
Trifolium arvense	30
Verbascum lychnitis	4
Verbascum nigrum	1
Veronica arvensis	2
Veronica chaemaedrys	11
Veronica officinalis	1
Vicia angustifolia	2
Vicia cracca	1
Vicia hirsuta	4
Vicia villosa	14
Viola arvensis	1
**Total**	2 016

Table S6. Correlation coefficients of all observed variables with impervious surfaces at three spatial scales; 100 m, 500 m and 1000 m. Highlighted are the highest absolute correlation coefficients.

	**Impervious surfaces 100 m**	**Impervious surfaces 500 m**	**Impervious surfaces 1000 m**
**Wild bees**			
Flower visits	-0.102	0.0325	0.125
Species richness	-0.332	-0.538	-0.511
Shannon diversity	-0.171	-0.598	-0.655
**Hoverflies**			
Flower visits	0.324	0.169	0.125
Species richness	0.001	-0.088	-0.022
Shannon diversity	-0.031	-0.071	0.026
**Butterflies**			
Flower visits	-0.568	-0.495	-0.333
Species richness	-0.699	-0.634	-0.480
Shannon diversity	-0.715	-0.600	-0.449
**Honeybee flower visits**	0.798	0.730	0.606
**Total pollinator flower visits**	0.351	0.382	0.389
**Flowering vegetation**			
Cover	0.648	0.735	0.726
Flower units	0.552	0.620	0.628
Shannon diversity	-0.609	-0.497	-0.400
Floral diversity	0.153	0.352	0.426
**Network metrics species level**			
Δ-d’	0.229	0.586	0.644
Δ-H2’	-0.401	-0.442	-0.386
Connectance species	-0.108	-0.286	-0.305
Δ-modularity	-0.349	-0.217	-0.130
NODF species	-0.134	-0.352	-0.427
**Network metrics genus level**			
Δ-d’	-0.160	0.283	0.359
Δ-H2’	-0.565	-0.472	-0.384
Δ-Connectance	0.019	-0.138	-0.152
Δ-modularity	-0.607	-0.455	-0.362
Δ-NODF	0.356	0.163	0.097

Table S7. List of plant species and their flower unit number estimated using quadrats.

Species	Flower units
Achillea millefolium s.l.	650
Anchusa officinalis	79
Arabidopsis thaliana	182
Arenaria serpyllifolia	99
Armeria maritima subsp. elongata	2
Berteroa incana	6497
Capsella bursa-pastoris	11
Cardamine hirsuta	15
Centaurea stoebe subsp. stoebe	154
Cerastium arvense	44
Cerastium semidecandrum	1824
Chondrilla juncea	3
Convolvulus arvensis	26
Crepis capillaris	27
Dianthus carthusianorum	45
Dianthus deltoides	22
Draba verna	71
Echium vulgare	594
Erigeron canadensis	130
Erodium cicutarium s. str	222
Euphorbia cyparissias	541
Galium verum	130
Geranium molle	2
Geranium pusillum	45
Helichrysum arenarium	272
Hypericum perforatum	11
Hypochaeris radicata	1
Jasione montana	312
Lamium purpureum	805
Medicago lupulina	12
Medicago x varia	1551
Myosotis ramosissima	475
Petrorhagia prolifera	29
Pilosella officinarum	8
Plantago lanceolata	158
Potentilla argentea	162
Potentilla verna	283
Saxifraga tridactylites	10
Scorzoneroides autumnalis	2
Sedum acre	304
Sedum sexangulare	19
Senecio vernalis	6
Spergula morisonii	2
Spergularia rubra	7
Tanacetum vulgare	124

**Species**	**Flower units**
Taraxacum sect. Ruderalia	19
Trifolium arvense	455
Verbascum lychnitis	49
Veronica arvensis	265
Vicia angustifolia	33
Vicia hirsuta	210
Vicia lathyroides	161
Vicia villosa	183
Viola arvensis	2

Table S8: Network metrics for each study site and taxonomic resolution (species and genus).

Site	Δ-d'	Δ-H2	Δ-modularity	Δ-connectance	Δ-NODF
**Species level**					
**1**	0.141	0.535	0.337	-0.065	-23.544
**2**	0.123	0.434	0.281	-0.046	-28.101
**3**	0.139	0.331	0.208	-0.049	-19.144
**4**	0.180	0.338	0.262	-0.075	-33.276
**5**	0.154	0.269	0.186	-0.039	-20.201
**6**	0.101	0.283	0.147	-0.032	-15.989
**7**	0.197	0.348	0.231	-0.028	-22.434
**8**	0.176	0.472	0.257	-0.044	-25.135
**9**	0.186	0.358	0.230	-0.067	-23.960
**10**	0.114	0.300	0.220	-0.029	-17.717
**11**	0.110	0.335	0.193	-0.042	-16.043
**Average**	0.147 (± 0.032 SD)	0.364 (± 0.078 SD)	0.232 (± 0.049 SD)	-0.047 (± 0.015 SD)	-22.322 (± 5.053 SD)
**Genus level**					
**1**	0.134	0.444	0.350	-0.093	-20.670
**2**	0.155	0.435	0.303	-0.057	-27.425
**3**	0.189	0.343	0.215	-0.075	-26.083
**4**	0.202	0.331	0.265	-0.102	-25.686
**5**	0.190	0.278	0.191	-0.045	-17.559
**6**	0.078	0.225	0.138	-0.037	-12.322
**7**	0.212	0.301	0.213	-0.038	-24.071
**8**	0.195	0.413	0.260	-0.065	-28.148
**9**	0.197	0.342	0.256	-0.109	-30.036
**10**	0.117	0.281	0.272	-0.044	-19.163
**11**	0.201	0.336	0.271	-0.073	-26.438
**Average**	0.170 (± 0.041 SD)	0.339 (± 0.066 SD)	0.249 (± 0.055 SD)	-0.067 (± 0.024 SD)	-23.418 (± 5.109 SD)

Supplementary Methods

The species identity of 47 bees that could not be identified unambiguously by morphological traits was confirmed by DNA barcoding. To do so, we extracted genomic DNA from one or two midlegs (depending on the size of the bee) using a Chelex extraction protocol[2]. The ca. 650 bp region of the mitochondrial cytochrome *c* oxidase subunit I (COI) gene was then amplified using DNA extracts with universal primers LCO-1490 (5'-GGTCAACAAATCATAAAGATATTGG-3') and HCO-2198 (5'-TAAACTTCAGGGTGACCAAAAAATCA-3')[3]. PCR reactions were carried out in 10 µL volumes consisting of 1 x PCR buffer containing 1.5 mM $MgCl_2$ (Promega, Madison, WI, USA), 200 µM of each dNTP, 0.4 µM of each primer, 1.5 U *Taq*-Polymerase (Promega) and 2 µL of template DNA (ca. 25-50 ng). PCRs were performed with a Biometra TProfessional basic gradient thermocycler (Biometra, Göttingen, Germany) under the following thermal regime: 3 min at 94°C, followed by 36 cycles of 30s at 94°C, 45s annealing at 50°C and 1 min at 72°C for elongation and a final elongation step at 72°C for 8 min. The PCR products were screened with a QiAxcel capillary electrophoresis system (Qiagen, Hilden, Germany) to confirm that a single PCR product of the correct size had been amplified. PCR products were purified using an ExoSAP-IT PCR Product Cleanup kit (Affymetrix, Santa Clara, CA, USA; Bell, 2008) and commercially sequenced on an ABI 3730xl DNA autosequencer with the LCO-1490 primer.

The resulting chromatograms were checked by eye, sequences were trimmed, manually aligned and checked for potential open reading frame shifts and premature stop codons in Geneious v.7.1.9 (https://www.geneious.com). Sequences were BLASTed against the NCBI GenBank nucleotide and the Barcode of Life (BOLD) databases[4]. We used a threshold BLAST hit similarity higher than 98 % for species identification.

References

1. QGIS Development Team. *QGIS Geographic Information System* (Open Source Geospatial Foundation Project, 2021).
2. Walsh, P. S., Metzger, D. A. & Higuchi, R. Chelex 100 as a medium for simple extraction of DNA for PCR-based typing from forensic material. *BioTechniques* **10,** 506–513 (1991).
3. Folmer, O., Black, M., Hoeh, W., Lutz, R. & Vrijenhoek, R. DNA primers for amplification of mitochondrial cytochrome c oxidase subunit I from diverse metazoan invertebrates. *Molecular marine biology and biotechnology* **3,** 294–299 (1994).
4. Ratnasingham, S. & Hebert, P. D. N. bold: The Barcode of Life Data System (http://www.barcodinglife.org). *Molecular ecology notes* **7,** 355–364; 10.1111/j.1471-8286.2007.01678.x (2007).
